# The Effect of BMI, Age, Gender, and Pubertal Stage on Bone Turnover Markers in Chinese Children and Adolescents

**DOI:** 10.3389/fendo.2022.880418

**Published:** 2022-06-13

**Authors:** Bingyan Cao, Meijuan Liu, Qipeng Luo, Qiao Wang, Min Liu, Xuejun Liang, Di Wu, Wenjing Li, Chang Su, Jiajia Chen, Chunxiu Gong

**Affiliations:** ^1^ Department of Endocrinology, Genetics and Metabolism, Beijing Children’s Hospital, Capital Medical University, National Center for Children’s Health, Beijing, China; ^2^ Department of Pain Medicine, Peking University Third Hospital, Beijing, China

**Keywords:** bone turnover markers (BTMs), body mass index (BMI), age, gender, pubertal stage, children

## Abstract

**Objectives:**

To ascertain the associations of serum bone turnover markers (BTMs) levels with body mass index (BMI) in Chinese children and adolescents, and whether the influence of BMI, age, pubertal stage on BTMs varied by gender.

**Methods:**

A total of 500 students (180 controls and 320 children and adolescents with overweight/obesity) aged 9–14 years were randomly selected from the Chinese National Survey on Students Constitution and Health Cohort. Serum levels of BTMs, including bone formation marker bone alkaline phosphatase (BAP), collagen type 1 C-terminal propeptide (CICP), and bone resorption markers C-terminal telopeptide of type-I collagen (CTX) were determined by commercial enzyme-linked immunosorbent assay kits. The associations among BMI, age, gender, pubertal stage, and BTMs were analyzed.

**Results:**

Serum levels of CICP and CTX in overweight/obese children and adolescents were lower than those in controls (*p*<0.05). Moreover, after subgroup analysis stratified by gender, the decreased serum CICP and CTX levels in overweight/obese children and adolescents were observed only in boys (*p*<0.05). After adjustment of age and pubertal stage, there was a negative correlation between serum BAP and BMI in both boys and girls (*p*<0.05). However, the correlations between serum CICP, CTX levels, and BMI were significant in boys but not in girls. Serum BAP and CICP levels were independently correlated with BMI, age, gender, and pubertal stage, while CTX levels were independently correlated with BMI, age, and gender (*p*<0.05). BAP, CICP, and CTX levels showed a clear age, gender, and pubertal stage dependence with significantly higher values in boys (*p*<0.05).

**Conclusions:**

Our findings support the associations between serum BTMs levels and BMI in Chinese children and adolescents, and suggest age, gender, and pubertal stage differences in this relationship that warrant future studies.

## Introduction

Childhood obesity [body mass index (BMI) ≥ 95 th percentile for age and gender] has become a global epidemic (1). Its worldwide prevalence has increased by 325% since the early 1960s (2). This issue poses a heavy economic burden on health systems and becomes a major health risk factor for individuals. Obesity-related metabolic diseases, such as hypertension (3), hyperlipidemia (4), coronary artery disease (5), insulin resistance (6), diabetes mellitus (7), and fatty liver disease (8) are the research hotspots in many published literature. Bone health has only recently drawn the attention of researchers.

Osteoporosis is a systemic skeletal disease, which is characterized by a deterioration of bone tissue microarchitecture that leads to low bone mineral density (BMD) and an increased risk of bone fractures (9). Osteoporosis affects more than 200 million people globally, with significant clinical and socio-economic implications (10). Osteoporosis used to occur mainly in adults but now occurs in children too (11). Peak bone mass, i.e. the highest BMD, is a key determinant of osteoporosis and fracture risk later in life (12). Childhood and adolescence are the most critical periods for bone mineral accrual, with peak bone mass being achieved around the age of 20 years. Epidemiologic data indicate that a 10% increase in peak bone mass in childhood is estimated to decrease the risk of osteoporotic fracture in the elderly by 50%, and the relative risk of fracture increases 2.6-fold for each 1 standard deviation (SD) decrease in bone mass (13). Therefore, understanding the characteristics of bone development in children and adolescents to achieve the ideal peak bone mass is pivotal for preventing osteoporosis and osteoporosis-related fractures.

Bone metabolism is a continuous remodeling or turnover process, which relies on the tightly coupled balance between bone formation by osteoblasts and bone resorption by osteoclasts. Children, with rapid bone growth and reshaping, have larger remodeling spaces and a shorter remodeling period than adults (14). Bon| turnover markers (BTMs) are a group of metabolites or enzymes which are released by osteoblasts or osteoclasts during the bone remodeling process. BTMs could provide a dynamic picture of changes in bone remodeling, and thus identify changes in the bone remodeling within a relatively short time interval (15). BTMs are independent predictors of fracture risk and future bone loss (16). The most studied BTMs are bone alkaline phosphatase (BAP), collagen type 1 C-terminal propeptide (CICP), reflecting bone formation by osteoblasts, and C-terminal telopeptide of type-I collagen (CTX), reflecting bone resorption by osteoclasts. Moreover, recent studies identified the bone as an endocrine organ, by producing and secreting “hormone-like factors” such as fibroblast growth factor 23 (FGF23) (17), which can affect bone remodeling and even the complete metabolism of the whole organism. BTMs and bone-derived factors levels could be affected by many factors, such as age, gender, pubertal stage, hormones, and nutritional status. Accurate measurement of BTMs and bone-derived factors are important for better understanding the connection between obesity and osteoporosis.

However, previous studies on serum BTMs and bone-derived factors levels in overweight/obese children provided conflicting results. For example, in an observational cross-sectional study involving 81 Italian children, Radetti et al. reported that serum CTX levels were significantly increased in obese children compared to controls (18). Furthermore, Dimitri et al. studied 103 United Kingdom (UK) children and demonstrated that serum CTX levels were significantly higher in obese children, even after correction for pubertal stage and sex (19). Otherwise, in a case-control study performed in 68 European children, Viljakainen et al. showed that obese subjects with early-onset severe obesity had significantly lower CTX levels than the sex- and age-matched controls (20).

As the data concerning serum BTMs and bone-derived factors levels in obese children were controversial, and few reported studies were performed in Chinese children, the objectives of the present study were (1) to evaluate serum BTMs and bone-derived factors levels in overweight/obese Chinese children and adolescents (2); to assess the associations between serum BTMs, bone-derived factors, and BMI (3); to explore the effects of age, sex, and pubertal stage on serum BTMs and bone-derived factors.

## Materials and Methods

### Participants and Study Design

This was a school-based cross-sectional study that was carried out in Beijing, the capital of China. Our study was a part of the Chinese National Survey on Students Constitution and Health. We screened the database and randomly selected 500 students aged 9–14 years, which were divided into two groups, the control group (n=180) and the overweight/obesity group (n=320). Children and adolescents were excluded if they had any of the following: chronic illness, metabolic bone disease, endocrine or known chromosomal abnormalities, or if they did not complete the questions in the questionnaires, or did not sign the informed consent. The ethics committee of Beijing Children’s Hospital, Capital Medical University approved this study, and all children’s parents signed the written informed consent.

### Anthropometric Measurements and Definitions

Anthropometric measurements including body height, body weight, and waist circumference (WC) were accomplished by the well-trained study assistants using uniform instruments. Body height (to the nearest 0.1 cm) was measured with subjects in the erect position without shoes (Seca 213 stadiometer, Hamburg, Germany), and weight (to the nearest 0.1 kg) was measured with subjects wearing light clothing (Tanita HA 503, Tanita Corporation, Tokyo, Japan). WC (to the nearest 0.1 cm) was obtained at the midpoint between the inferior costal margin and the superior border of the iliac crest on the midaxillary line (WT-21, Wintape, Guangdong, China). Two measurements (measurement error ≤ 0.1 cm) were recorded and the average was used for the analysis. BMI (kg/m^2^) was calculated as weight (kg) divided by height squared (m^2^). According to age- and sex-specific BMI cut-off points recommended by the Working Group for Obesity in China, overweight was defined as BMI between the 85 th and 95 th percentile, while obesity was defined as BMI ≥95 th percentile (21). Puberty was assessed by the well-trained study assistants using the standardized method of Tanner stages (22).

### Questionnaire

Information about each subject regarding demographic and lifestyle factors was collected by the trained investigators through a structured parent questionnaire (23, 24). Children’s lifestyle habits were ascertained on the questionnaire by asking “On average, how many times a week does your child exercise? (≤3 times/week or >3 times/week) “, “How many minutes per week does your child exercise? (≤120 minutes/week or >120 minutes/week) “, “How many hours per week does your child spend watching television (TV)? (≤2 hours/day or >2 hours/day)”, “On most nights, how many hours does your child sleep each night? (<9 hours/day or ≥9 hours/day)”.

### Blood Sample Collection and BTMs, FGF23 Assays

Venous blood samples were taken from all subjects after an overnight fast. According to the manufacturer’s instructions, serum BAP was determined using an immune-enzymatic assay (Octeia Ostase BAP, IDS ltd, Baldon, UK). The lower limit of detection was 0.7 ug/L. Serum CICP was analyzed using an enzyme-linked immunoadsorbent assay (ELISA) with a Metra CICP EIA kit (Quidel Corporation, San Diego, CA, USA). The lower limit of detection was 0.02 ng/mL. Serum CTX was measured using the IDS-iSYS CTX (CrossLaps^®^) assay (Immunodiagnostic Systems, plc, Tyne and Wear, UK). The lower limit of detection was 0.2 ng/mL. Serum FGF23 was tested by a commercially available ELISA kit (Wuhan Cusabio Biotech Co., Ltd., Wuhan, China). The minimum detectable concentration was 0.78 pg/mL.

### Statistical Analysis

Values were expressed as mean ± SD for continuous variables, and as numbers (percentages) for categorical variables. Comparisons for continuous and categorical variables were made by the independent t-test and chi-square test, respectively. Spearman partial correlation coefficients were used to describe the associations between BTMs, FGF23, and BMI. Stepwise multiple regression analysis was executed to explore the variables independently related to serum BTMs and FGF23 levels. The statistical analyses were achieved using SPSS version 20.0 for Windows (SPSS Inc, Chicago, IL, USA) and a *p*-value <0.05 was considered statistically significant.

## Results

### Serum BTMs, FGF23 Levels, and Other Characteristics in the Study Population

**Table 1** presented the baseline characteristics, serum BTMs and FGF23 levels of all subjects. The lifestyle characteristics of all participants were shown in **Supplementary Table 1**. Overweight/obese children had significantly higher body weight, BMI, and WC than controls (*p*<0.05), regardless of their gender (*p*<0.05). The proportion of girls presenting in the advanced Tanner stages (stage III-V) was greater than that of boys, both in the control group and the overweight/obesity group (*p*<0.05). Serum levels of CICP and CTX in overweight/obese children were lower than that in controls (*p*<0.05). Moreover, after subgroup analysis stratified by gender, the decreased serum CICP and CTX levels in overweight/obese children were observed only in boys (*p*<0.05). Serum BAP and FGF23 levels were not different between the control group and the overweight/obesity group in both genders (*p*>0.05). Boys had higher levels of BAP, CICP as well as CTX than girls, both in the control group and the overweight/obesity group (*p*<0.05). However, no difference was detected between boys and girls in FGF23 levels (*p*>0.05).

**Table 1:** Comparison of subjects with and without overweight/obesity according to age, anthropometric measures, Tanner stage, and bone turnover markers of both sexes.

Parameters	Overall cohorts (n=500)			Control group (n=180)			Overweight/obesity group (n=320)		
	All (n=500)	Boys (n=297)	Girls (n=203)	All (n=180)	Boys (n=109)	Girls (n=71)	All (n=320)	Boys (n=188)	Girls (n=132)
**Age (years)**	12.18 ± 1.79	12.22 ± 1.72	12.13 ± 1.89	12.33 ± 1.74	12.33 ± 1.68	12.33 ± 1.84	12.10 ± 1.82	12.15 ± 1.75	12.02 ± 1.92
**Body weight (kg)**	61.00 ± 20.82	63.16 ± 22.18	57.85 ± 18.24^b^	44.73 ± 10.75	45.47 ± 11.10	43.60 ± 10.17	70.16 ± 19.48^a^	73.42 ± 20.48^e^	65.51 ± 16.98 ^df^
**Body height (cm)**	157.79 ± 12.81	159.93 ± 13.53	154.65 ± 10.97^b^	156.33 ± 12.44	157.59 ± 13.35	154.39 ± 10.70	158.61 ± 12.96	161.28 ± 13.48^e^	154.80 ± 11.15^d^
**BMI (kg/m^2^)**	23.99 ± 5.98	24.11 ± 6.03	23.81 ± 5.90	17.99 ± 2.16	17.97 ± 2.02	18.00 ± 2.38	27.36 ± 4.64^a^	27.66 ± 4.55^e^	26.94 ± 4.74^f^
**WC (cm)**	76.67 ± 14.52	79.31 ± 15.15	72.80 ± 12.62^b^	63.69 ± 6.41	64.58 ± 6.39	62.32 ± 6.25^c^	83.96 ± 12.57^a^	87.85 ± 11.82^e^	78.44 ± 11.54^df^
**Tanner stage**									
**I**	108 (21.6%)	92 (31.0%)	16 (7.9%)^b^	38 (21.1%)	30 (27.5%)	8 (11.3%)^c^	70 (21.9%)	62 (33.0%)	8 (6.1%)^d^
**II**	38 (7.6%)	27 (9.1%)	11 (5.4%)	16 (8.9%)	9 (8.3%)	7 (9.9%)	22 (6.9%)	18 (9.6%)	4 (3.0%)
**III**	121 (24.2%)	67 (22.6%)	54 (26.6%)	42 (23.3%)	27 (24.8%)	15 (21.1%)	79 (24.7%)	40 (21.3%)	39 (29.5%)
**IV**	119 (23.8%)	48 (16.2%)	71 (35.0%)	47 (26.1%)	21 (19.3%)	26 (36.6%)	72 (22.5%)	27 (14.4%)	45 (34.1%)
**V**	114 (22.8%)	63 (21.2%)	51 (25.1%)	37 (20.6%)	22 (20.2%)	15 (21.1%)	77 (24.1%)	41 (21.8%)	36 (27.3%)
**BAP (ug/L)**	97.90 ± 45.50	110.38 ± 39.48	79.64 ± 47.61^b^	100.66 ± 46.71	113.45 ± 41.81	81.02 ± 47.31^c^	96.35 ± 44.80	108.60 ± 38.07	78.90 ± 47.93^d^
**CICP (ng/mL)**	256.23 ± 132.96	293.19 ± 127.15	202.15 ± 122.63^b^	274.42 ± 145.85	315.11 ± 137.60	211.95 ± 136.57^c^	246.00 ± 124.20^a^	280.48 ± 119.21^e^	196.88 ± 114.63^d^
**CTX (ng/mL)**	1.62 ± 0.74	1.85 ± 0.70	1.29 ± 0.66^b^	1.73 ± 0.81	2.00 ± 0.80	1.30 ± 0.64^c^	1.56 ± 0.69^a^	1.76 ± 0.63^e^	1.28 ± 0.67^d^
**FGF23 (pg/mL)**	51.60 ± 69.75	54.29 ± 70.95	47.68 ± 67.94	50.22 ± 64.68	51.80 ± 64.67	47.79 ± 65.38	52.39 ± 72.53	55.73 ± 74.57	47.62 ± 69.52

BMI, body mass index; WC, waist circumstance; TV, television; BAP, bone-specific alkaline phosphatase; CICP, C-propeptide of type I procollagen; CTX, collagen type 1 C-terminal propeptide; FGF23, fibroblast growth factor 23. Values were presented as N (%) or mean ± SD as appropriate. P values were obtained by Student’s t-test or chi-square test. ^a^P < 0.05 between control group and overweight/obesity group in the overall cohorts; ^b^P < 0.05 between boys and girls in the overall cohorts; ^c^P < 0.05 between boys and girls in the control group; ^d^P < 0.05 between boys and girls in the overweight/obesity group; ^e^P < 0.05 between control group and overweight/obesity group in boys; ^f^P < 0.05 between control group and overweight/obesity group in girls.

### Association of BTMs, FGF23 with BMI

**Figure 1:**
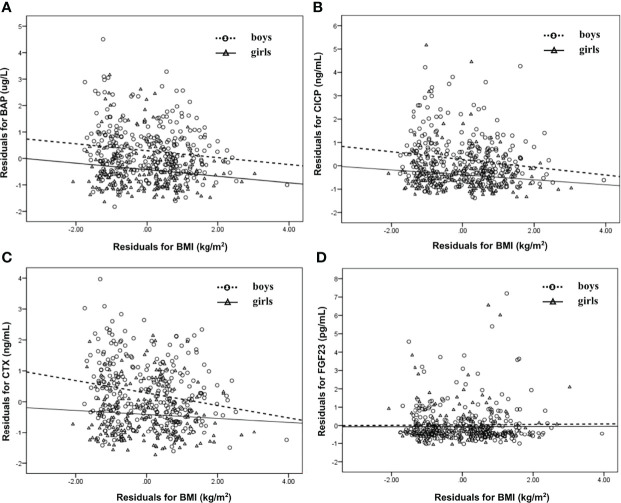
Scatter plot that showing the relationship between BAP **(A)**, CICP **(B)**, CTX **(C)**, FGF23 **(D)** and BMI in boys and girls, respectively. Partial correlation analysis was performed after adjustment for age and pubertal stages. BAP, bone-specific alkaline phosphatase; CICP, C-propeptide of type I procollagen; CTX, collagen type 1 C-terminal propeptide; FGF23, fibroblast growth factor 23; BMI, body mass index.

The associations of serum BTMs and FGF23 levels with BMI were studied separately for boys and girls. As illustrated in **Figure 1A**, when age and pubertal stage were taken into account, serum BAP levels were negatively correlated with BMI in both genders (r=−0.130 for BAP in boys, r=−0.164 for BAP in girls, *p* all<0.05). After adjusting for age and pubertal stage, serum CICP and CTX levels were negatively associated with BMI only amomg boys (r=−0.162 for CICP in boys, r=−0.200 for CTX in boys, *p* all<0.05; r=−0.127 for CICP in girls, r=−0.069 for CTX in girls, *p* all>0.05) (**Figures 1B, C**). There was no significant relationship between serum FGF23 levels and BMI neither in boys nor in girls (*p*>0.05).

Next, stepwise multivariate linear regression was performed. As displayed in **Table 2**, BMI, age, sex, pubertal stage, and frequency of exercise were independently associated with serum BAP levels after adjusting for WC, exercise time, watching TV, and sleep duration. Among them, BMI was independently and negatively related to serum BAP levels (β=-0.130, *p*<0.05), which agreed with the results shown in **Figure 1** by partial correlation analysis. After adjusting for WC, exercise time, watching TV, and sleep duration, BMI, age, sex, and pubertal stage were independent factors associated with serum CICP levels, while BMI, age, and sex were independent factors associated with serum CTX levels. Moreover, serum CICP and CTX were also independently and negatively correlated with BMI (β=-0.151 for CICP, β=-0.152 for CTX, *p* all<0.05), which was consistent with the results demonstrated in **Table 1**. In addition, age was independently and negatively related to FGF23 levels after controlling for other confounders.

**Table 2:** Multiple regression analysis for the variables independently related to serum BTMs and FGF23 levels in all subjects.

	B	se of B	β	*p*
Serum BAP (R^2^ = 0.301)				
BMI	-0.993	0.300	-0.130	<0.05
Age	-12.023	1.595	-0.474	<0.05
Sex	-36.538	3.842	-0.395	<0.05
Pubertal stage	5.982	1.987	0.189	<0.05
Frequency of exercise	-8.084	3.789	-0.089	<0.05
(Constant)	312.404	17.157		<0.05
Serum CICP (R^2^ = 0.174)				
BMI	-3.349	0.951	-0.151	<0.05
Age	-20.093	4.900	-0.271	<0.05
Sex	-104.680	12.162	-0.387	<0.05
Pubertal stage	14.314	6.305	0.154	<0.05
(Constant)	682.889	54.467	12.538	
Serum CTX (R^2^ = 0.181)				
BMI	-0.019	0.005	-0.152	<0.05
Age	-0.046	0.018	-0.112	<0.05
Sex	-0.572	0.061	-0.380	<0.05
(Constant)	3.441	0.234	14.720	<0.05
Serum FGF23 (R^2^ = 0.042)				
Age	-8.108	1.703	-0.209	<0.05
(Constant)	150.369	20.972		<0.05

Variables also entered multiple regression analysis but not included in the equation: waist circumstance, exercise time, watching TV, sleep duration. R^2^, multiple determination coefficient; B, unstandardized regression coefficient; se of B, standard error of the unstandardized regression coefficient; β, standardized regression coefficient.

### Association of BTMs, FGF23 with Age

**Figure 2:**
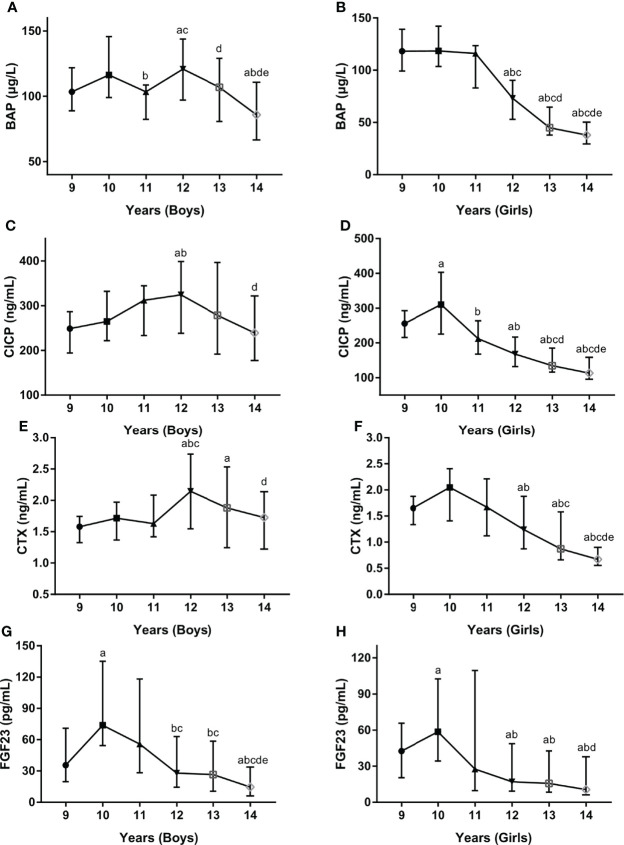
Serum levels for BAP **(A**, **B)**, CICP **(C**, **D)**, CTX **(E**, **F)**, FGF23 **(G**, **H)** by age and gender. BAP, bone-specific alkaline phosphatase; CICP, C-propeptide of type I procollagen; CTX, collagen type 1 C-terminal propeptide; FGF23, fibroblast growth factor 23. ^a^P < 0.05 compared with children aged 9 years; ^b^P < 0.05 compared with children aged 10 years; ^c^P < 0.05 compared with children aged 11 years; ^d^P < 0.05 compared with children aged 12 years; ^e^P < 0.05 compared with children aged 13 years.

Since age was found to be an independent influence factor for serum BTMs levels, serum levels of BTMs and FGF23 were further examined in different age groups stratified by gender. In boys, serum BAP levels fluctuated with age, with a nadir value at 14 years (93.64 ug/L) and a peak value at 12 years (126.68 ug/L) (**Figure 2A**). In girls, BAP started with high values at 9 years of age (125.66 ug/L) and subsequently decreased until reaching a nadir at 14 years of age (42.61 ug/L) (**Figure 2B**). In boys, CICP levels were low at 9 years (248.08 ng/mL), then revealed a clear tendency to increase until 12 years (344.13 ng/mL) and followed by decreasing values after 12 years (**Figure 2C**). In girls, CICP started with high values at 9 years (274.61 ng/mL) and reached a peak value at 10 years (343.85 ng/mL), and subsequently decreased until reaching a nadir at 14 years (128.98 ng/mL) (**Figure 2D**). Serum CTX levels also showed similar trends, with a peak value at age 12 in boys (2.14 ng/mL) and at age 10 (1.95 ng/mL) in girls, a nadir at age 9 in boys (1.60 ng/mL) and age 14 in girls (0.78 ng/mL) (**Figures 2E, F**). Serum FGF23 levels exhibited similar trends in both sexes across all age groups, with a peak value at 10 years (115.25 pg/mL for boys, 79.01 pg/mL for girls) and a nadir at 14 years (33.81 pg/mL for boys, 26.96 pg/mL for girls) (**Figures 2G, H**).

### Association of BTMs, FGF23 with Pubertal Stage

**Figure 3:**
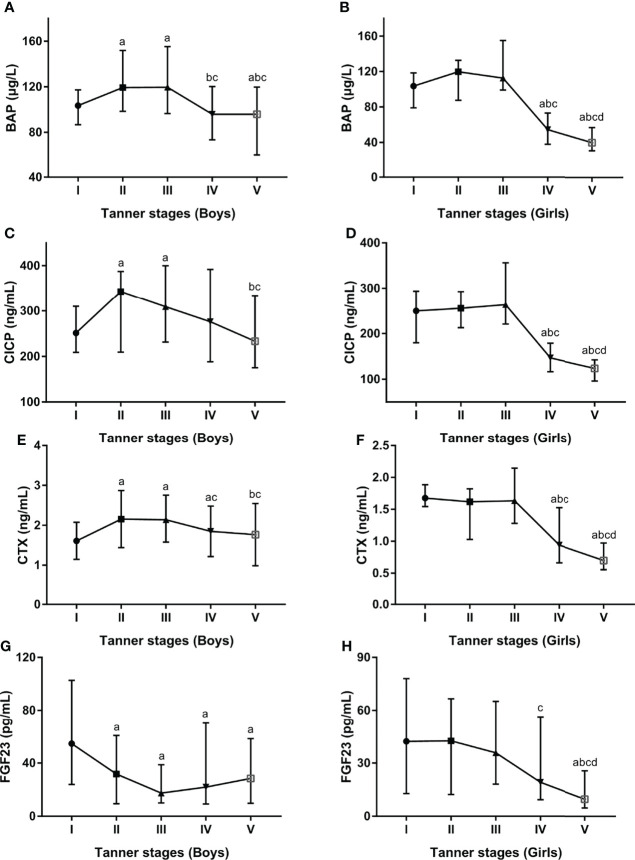
Serum levels for BAP **(A**, **B)**, CICP **(C**, **D)**, CTX **(E**, **F)**, FGF23 **(G**, **H)** by developmental staging and gender. BAP, bone-specific alkaline phosphatase; CICP, C-propeptide of type I procollagen; CTX, collagen type 1 C-terminal propeptide; FGF23, fibroblast growth factor 23. ^a^P < 0.05 compared with Tanner stage I; ^b^P < 0.05 compared with Tanner stage II; ^c^P < 0.05 compared with Tanner stage III; ^d^P < 0.05 compared with Tanner stage IV.

Additionally, serum BTMs and FGF23 concentrations were further explored in different pubertal stages stratified by gender. In boys, serum BTMs levels presented similar trends across Tanner stages, with peak values at Tanner stages II and III and nadir levels at Tanner stages IV and V (**Figures 3A, C, E**). In girls, serum BTMs levels have also shown similar trends with Tanner stages. Serum BTMs levels started with high values at Tanner stage I, remained relatively constant in Tanner stages II-III, and then decreased until reaching a nadir at Tanner stage V (**Figures 3B, D, F**). For FGF23 levels, the trends among boys and girls at different Tanner stages were quite different. In boys, FGF23 peaked at Tanner stage I, then decreased until reaching a nadir at Tanner stage III, followed by increasing values after Tanner stage III (**Figure 3G**). In girls, FGF23 started with high values at Tanner stage I, and subsequently decreased until reaching a nadir at Tanner stage V (**Figure 3H**).

## Discussion

Obesity is closely associated with impaired bone homeostasis. BTMs, which reflect the dynamic changes in bone remodeling, are sensitive indicators of early bone metabolism disturbances. Although many studies have examined the associations between serum BTMs and BMI in children and adolescents, the results were inconsistent and were largely based on foreign studies (18–20, 25–31). To date, this is the first study to investigate serum BTMs levels in Chinese overweight/obese children and adolescents.

In the current study, we showed that serum levels of CICP (a marker of bone formation) and CTX (a marker of bone resorption) were lower in overweight/obese children than in controls. This is in agreement with a recent study among children in Europe, which demonstrated that all BTMs, except BAP, were lower in obese children compared with the age-and sex-matched controls (20). Similarly, in the USA, Reinehr et al. observed significantly lower circulating osteocalcin (OC, a marker of bone formation) levels in obese children than in normal-weight children (25). Moreover, after one-year lifestyle intervention, serum OC levels in obese children with substantial weight loss were significantly increased (25). However, there are also several studies presenting inconsistent results. In both the studies conducted in Italy (18) and the UK (19), serum CTX levels were significantly increased in obese children compared to controls. There are several reasons for inconsistent results in the different studies. Firstly, Radetti et al. recruited 81 children, which were further divided into the overweight/obese group (n=44) and lean group (n=37), the small sample size of fewer than 50 subjects in each group (18) may cause false positives and low power. Secondly, Dimitri et al. explored serum BTMs levels in obese children, particularly in those who have fractured, the high proportion of children with previous fractures (26/103) (19) may also affect the results of serum BTMs. Thirdly, race differences may be also an important factor. In brief, previous studies on serum BTMs in overweight/obese children were mainly carried out in Caucasian populations, our study showed decreased serum BTMs levels in Chinese overweight/obese children.

As we all know, obese children often manifested an earlier onset of puberty in comparison with children with normal weight (32), which was also observed in our study. We then explored the associations of serum BTMs and BMI after adjusting the age and pubertal Tanner stage, we found that all BTMs, including BAP, CICP, and CTX had a negative correlation with BMI. Moreover, multivariate linear regression showed that all serum BTMs were independently and negatively associated with BMI after controlling for other confounders. Similarly, Geserick et al. showed that age- and gender-adjusted BTMs were significantly lower in obese children than in controls independent of their pubertal development (27). Thus, all these findings together with our results suggest the negative associations between serum BTMs and BMI. According to previous studies, the decreased process of bone turnover in overweight/obese children could influence BMD, bone quality, and bone strength, and consequently increased the risk of fracture (33). However, given the cross-sectional design of this study, the directionality of the association between serum BTMs and BMI cannot be conclusively established.

In addition, our study observed a clear gender difference in serum BTMs. Previously, Mayer et al. studied 397 German children with a wide range of BMI (217 subjects without obesity, 180 subjects with obesity) and reported significantly higher bone formation as well as resorption markers in boys than in girls (34). In our present study, higher levels of serum BTMs in boys were observed both in the control and overweight/obese groups. The higher serum BTMs in boys may be attributed to physical factors (higher physical activity and muscle strength in boys) and biological factors (gender hormones) (34). As a result of testosterone acting on periosteal apposition, boys have a greater width and size of bones than girls of the same age (35).

Interestingly, the correlations between serum BTMs and BMI were more pronounced in boys. On the one hand, the decreased serum CICP and CTX levels in overweight/obese children were observed only in boys. On the other hand, the correlations between serum CICP, CTX levels, and BMI were significant in boys but not in girls. These findings suggest that gender differences should be considered by local policy-makers when designing initiatives to address issues around bone health in children with overweight/obesity. Further studies are needed to uncover the underlying mechanism. Although we are unclear about what may drive the gender differences, the interconnections of adipokines, BTMs, and sex hormones warrant further investigation.

Apart from gender, age and puberty also were the significant and independent determinants of serum BTMs levels, which was expected given the growth and development present during childhood and adolescence. Interestingly, when both age and gender were considered simultaneously, gender demonstrated different effects in different age bands. In girls, serum BTMs showed a peak at 10 years of age and then dropped rapidly thereafter, while in boys peak value of those biomarkers was observed at 12 years of age. These results were similar to previous studies conducted in Finish (girls, peak levels at 11 years; boys, peak levels at 14 years) (36), German (girls, peak levels at 10 to 11 years; boys, peak levels at 13 years) (27), and Polish (girls, peak levels at 8 to 13 years; boys, peak levels at 10 to 15 years) (37) children and adolescents. When both puberty and gender were considered simultaneously, serum BTMs levels presented similar trends across Tanner stages in boys and girls, with peak values at Tanner stages II and III and nadir levels at Tanner stages IV and V. Consistent with our findings, a cross-sectional study conducted by Bayer et al. among 439 Caucasian children reported the peak values of serum OC and procollagen type I N-terminal propeptide with the pubertal growth spurt at second-third Tanner stages (38). As age and pubertal stage are closely linked, older age of onset of puberty was associated with later peak values of serum BTMs observed in boys. However, given our present study was conducted in children and adolescents aged 9–14 years, further research is required on the influence of age, gender, and pubertal stages on serum BTMs, in particular in different age groups.

In recent research, the bone has been identified as an important endocrine organ. FGF23, one of the bone-derived factors, plays an important role not only in bone homeostasis but also in regulating whole-body energy metabolism. However, the data from existing research on serum FGF23 levels in children with obesity are inconsistent. Some studies reported an increase in serum FGF-23 concentration in overweight/obese children (39, 40) and a positive association between serum FGF23 and BMI (39). In contrast, some studies detected decreased serum FGF-23 levels in obese subjects (41) and inverse correlations with fasting insulin levels (41) as well as fasting glucose levels (42). In our present study, we did not detect a significant difference in serum FGF-23 levels between the overweight/obese group and the control group. Noteworthy, we found that age was independently and negatively related to FGF23 levels after controlling for other confounders. Our findings were in line with a previous study in German children and adolescents that showed serum FGF-23 was significantly correlated with age (43). However, contrary to that study, we also found that when both puberty and gender were considered at the same time, the trends for serum FGF23 levels across Tanner stages were quite different between boys and girls. Thus, future studies are needed to have a better understanding of the association between serum FGF23 and BMI in age-gender-puberty populations.

This study has some limitations that should be taken into consideration. Firstly, since it was a cross-sectional study, the causality cannot be determined. Secondly, there was a large difference in the number of respondents between the two groups, which probably affected the results obtained in our study. Thirdly, BMD, bone mineral content, and bone areas were not assessed in our study. Fourthly, in terms of external validity, this was a single-center study with a relatively small sample size, our results cannot be extrapolated to the whole country.

In conclusion, the present study revealed that serum BTMs levels were significantly decreased in overweight/obese Chinese children and adolescents, and were independently negatively associated with BMI. Furthermore, serum BTMs levels showed a clear age, gender, and pubertal stage dependence with significantly higher values in boys. Future studies should be performed to establish age- gender- and puberty-dependent references of serum BTMs levels in Chinese children.

## Data Availability Statement

The raw data supporting the conclusions of this article will be made available by the authors, without undue reservation.

## Ethics Statement

The studies involving human participants were reviewed and approved by The ethics committee of Beijing Children’s Hospital, Capital Medical University approved this study, and all children’s parents signed the written informed consent. Written informed consent to participate in this study was provided by the participants’ legal guardian/next of kin.

## Author Contributions

All authors helped to perform the research; BC contributed to the project management; MJL wrote the manuscript; QL participated in the interpretation of data; QW, ML, XL, DW, WL, CS, and JC took part in the collection of clinical samples; CG conceived and designed the project as well as revised the manuscript. All listed authors revised the paper critically and approved the final version of the submitted manuscript.

## Funding

This study was funded by The Pediatric Medical Coordinated Development Center of Beijing Hospitals Authority (XTYB201808), the National Key Research and Development Program of China (2016YFC0901505, 2016YFC1305304), and the Beijing Municipal Administration of Hospital Clinical Medicine Development of Special Funding Support (No. ZYLX201821).

## Conflict of Interest

The authors declare that the research was conducted in the absence of any commercial or financial relationships that could be construed as a potential conflict of interest.

## Publisher’s Note

All claims expressed in this article are solely those of the authors and do not necessarily represent those of their affiliated organizations, or those of the publisher, the editors and the reviewers. Any product that may be evaluated in this article, or claim that may be made by its manufacturer, is not guaranteed or endorsed by the publisher.
