# Supplementary material for: The Effect of BMI, Age, Gender, and Pubertal Stage on Bone Turnover Markers in Chinese Children and Adolescents
Source: Front Endocrinol (Lausanne). 2022 Jun 13;13:880418. doi: 10.3389/fendo.2022.880418 (PMC9234688; doi:10.3389/fendo.2022.880418)
Supplement: Supplementary file 1 [file Table_1.docx]

**Supplementary Table 1 Lifestyle characteristics of the study participants.**

| **Parameters** | **Overall cohorts (n=500)** | | | **Control group (n=180)** | | | **Overweight/obesity group (n=320)** | | |
| --- | --- | --- | --- | --- | --- | --- | --- | --- | --- |
|  | **All (n=500)** | **Boys (n=297)** | **Girls (n=203)** | **All (n=180)** | **Boys (n=109)** | **Girls (n=71)** | **All (n=320)** | **Boys (n=188)** | **Girls (n=132)** |
| **Frequency of exercise** |  |  |  |  |  |  |  |  |  |
| **≦3 times/week** | 262 (52.4%) | 157 (52.9%) | 105 (51.7%) | 89 (49.4%) | 57 (52.3%) | 32 (45.1%) | 173 (54.1%) | 100 (53.2%) | 73 (55.3%) |
| **>3 times/week** | 238 (47.6%) | 140 (47.1%) | 98 (48.3%) | 91 (50.6%) | 52 (47.7%) | 39 (54.9%) | 147 (45.9%) | 88 (46.8%) | 59 (44.7%) |
| **Exercise time** |  |  |  |  |  |  |  |  |  |
| **≦120 minutes/week** | 286 (57.2%) | 170 (57.2%) | 116 (57.1%) | 95 (52.8%) | 57 (52.3%) | 38 (53.5%) | 191 (59.7%) | 113 (60.1%) | 78 (59.1%) |
| **>120 minutes/week** | 214 (42.8%) | 127 (42.8%) | 87 (42.9%) | 85 (47.2%) | 52 (47.7%) | 33 (46.5%) | 129 (40.3%) | 75 (39.9%) | 54 (40.9%) |
| **Watching TV** |  |  |  |  |  |  |  |  |  |
| **<****2 hours/day** | 387 (77.4%) | 232 (78.1%) | 155 (76.4%) | 137 (76.1%) | 85 (78.0%) | 52 (73.2%) | 250 (78.1%) | 147 (78.2%) | 103 (78.0%) |
| **≥2 hours/day** | 113 (22.6%) | 65 (21.9%) | 48 (23.6%) | 43 (23.9%) | 24 (22.0%) | 19 (26.8%) | 70 (21.9%) | 41 (21.8%) | 29 (22.0%) |
| **Sleep duration** |  |  |  |  |  |  |  |  |  |
| **<9 hours/day** | 400 (80.0%) | 234 (78.8%) | 166 (81.8%) | 144 (80.0%) | 85 (78.0%) | 59 (83.1%) | 256 (80.0%) | 149 (79.3%) | 107 (81.1%) |
| **≥9 hours/day** | 100 (20.0%) | 63 (21.2%) | 37 (18.2%) | 36 (20.0%) | 23 (22.0%) | 12 (16.9%) | 64 (20.0%) | 39 (20.7%) | 25 (18.9%) |

Values were presented as N (%).
